# Design optimization and experimental evaluation of a large capacity magnetorheological damper with annular and radial fluid gaps

**DOI:** 10.1177/1045389X221151075

**Published:** 2023-01-21

**Authors:** Moustafa Abdalaziz, Ramin Sedaghati, Hossein Vatandoost

**Affiliations:** Department of Mechanical, Industrial and Aerospace Engineering, Concordia University, Montreal, QC, Canada

**Keywords:** Magnetorheological damper, bypass MR valve, design optimization, performance evaluation

## Abstract

This paper presents an optimal design of a large-capacity Magnetorheological (MR) damper suitable for off-road vehicle applications. The damper includes an MR fluid bypass valve with both annular and radial gaps to generate a large damping force and dynamic range. An analytical model of the proposed damper is formulated based on the Bingham plastic model of MR fluids. To establish a relationship between the applied current and magnetic flux density in the MR fluid active regions, an analytical magnetic circuit is formulated and further compared with a magnetic finite element model. The MR valve geometrical parameters are subsequently optimized to maximize the damper dynamic range under specific volume and magnetic field constraints. The optimized MR valve can theoretically generate off-state and on-state damping forces of 1.1 and 7.41 kN, respectively at 12.5 mm/s damper piston velocity. The proposed damper has been also designed to allow a large piston stroke of 180 mm. The proof-of-concept of the optimally designed MR damper was subsequently fabricated and experimentally characterized to investigate its performance and validate the models. The results show that the proposed MR damper is able to provide large damping forces with a high dynamic range under different excitation conditions.

## 1. Introduction

MR fluids are smart controllable materials whose rheological properties, such as apparent viscosity and yield strength, can be instantly varied by applying an external magnetic field. The MR fluid is basically composed of micron-sized ferromagnetic iron particles such as carbonyl iron particles (CIP) dispersed in a carrier liquid such as silicon oil. Surfactants are generally being added to the carrier fluids in order to reduce the sedimentation of the heavy magnetic particles within the liquid medium and also to enhance the distribution of the particles in the carrier fluid (Munoz et al., 2001; Park and Park, 2001). MR fluids have been employed in many adaptive devices due to their rapid response time, fail-safe feature, and low power consumption (Carlson and Jolly, 2000). The MR fluid-based devices can operate under different operating modes such as valve mode, shear mode, squeeze mode, or a combination of them (Butz and Von Stryk, 2002; Carlson and Jolly, 2000; Olabi and Grunwald, 2007). The typical operating mode in MR fluid-based valves and dampers is the valve mode. These valves/dampers should possess unique properties such as design simplicity, high damping force, extensive dynamic force range, damping variability, and fast response. Therefore, they can be effectively utilized to mitigate vibration in a broad-band frequency range.

The MR fluid valves typically consist of a magneto-conductive body with an embedded electromagnet which can magnetically activate the MR fluid during passage through a valve orifice. This will yield variation in the fluid viscosity that results in resistance to the fluid flow (Manjeet and Sujatha, 2019). The MR fluid valve is typically fitted internally in the MR damper or externally, so-called as a MR fluid bypass valve. The configuration of valve gaps can be annular, radial, or a combination of both. The geometrical structure of MR valves can significantly impact its dynamic performance, thus optimizing the valve’s geometrical structure is a crucial part of designing MR valves, irrespective of their application. For instance, the gap size has been identified as an important parameter to be optimized as it significantly affects the dynamic performance of the MR valves (Hu et al., 2017, 2018). Wider gaps generally reduce viscous and controllable yield damping forces, while a smaller gap increases the pressure drop but it may also cause valve blockage. Practically, the gap size ranges between 0.5 and 2 mm (Ai et al., 2006; Fatah et al., 2015; Hu et al., 2018), depending on the design requirements.

At the early design stages of MR dampers, the Bingham plastic model based on quasi-static fluid behavior has been extensively used to predict the damping force as a function of applied currents (Parlak and Engin, 2012). Later in the design process, phenomenological or parametric dynamic models can be applied to predict the damping forces based on the magnetic field, excitation frequency, and displacement amplitude. Significant research studies have been conducted on the design and optimization of MR valves to maximize dynamic range (high on-state and low off-state damping forces) while minimizing the time response (Abouobaia et al., 2020; Nguyen and Choi, 2009; Nguyen et al., 2008). Rosenfeld and Wereley (2004), for instance, presented a design strategy guideline for analytical optimization under constrained volumes based on assuming a constant magnetic flux through the magnetic circuit. This assumption may result in suboptimal results since the MR valve performance depends on the magnetic circuit as well as the geometrical dimension of the valve. Nguyen et al. (2008) introduced an optimal design for the determination of geometrical dimensions of MR valves featuring annular configurations to minimize the electromagnetic coil energy consumption and time response. The golden-section algorithm and local quadratic fitting technique were used as optimization methods to find the optimum solution. The results asserted the importance of the optimal design of the MR valve structural parameters. It was also reported that the wire diameter has less importance and thus can be neglected in minimizing power consumption. Nguyen et al. (2009) later presented analytical design optimization for the identification of geometrical parameters of single and double coil annular MR valves that are constrained in a specific volume to maximize the controllable pressure drop. The optimum analytical solution was verified with a finite element optimal solution. The error between the two methods was reported to be less than 7%. Hadadian et al. (2014) proposed an optimum design strategy for an annular MR valve with a single-coil constrained in a specific volume. They developed smooth response surface functions for magnetic field intensity in active MR fluid region using the response surface method and design of experiments. The genetic algorithm (GA) and the sequential quadratic programming (SQP) methodology were used to capture the global optimum solution.

Most of the above-mentioned studies have conducted design optimization of MR valves based on annular gap configuration. However, enhancing the valve dynamic performance generally requires increasing the annular gap length or increasing the number of coils which both substantially increase the MR valve size, complicity, and cost. The annular-radial integration design may overcome the limitations associated with the annular-gap design. In a few studies, the integrated annular-radial gap design has been successfully applied to the development of high-performance MR valves (Bai et al., 2013; Ichwan et al., 2016). Hu et al. (2014), for instance, developed an MR fluid valve with a tunable conical annular gap between 1–2 mm by rotating the valve spool to increase the pressure drop without enlarging the valve size or consuming more power. The experimental results showed that the pressure drop could fluctuate between 130 and 1150 kPa. They later proposed an annular-radial MR valve with variable radial gaps to meet different working conditions (Hu et al., 2017). The gap variability has been achieved by replacing washers with different thicknesses. A maximum damping force of 4.72 kN at applied current 2 A and a dynamic range of nearly 7 were achieved. Imaduddin et al. (2015) proposed an MR valve with multiple annular and radial gaps in order to extend the length of MR fluid active region. Also, the effect of the gap size on the pressure drop was discussed. The experimental results reported that the valve could cause a pressure drop of more than 2.5 MPa.

Although the reported studies focusing on designing MR valves with annular-radial gap confirmed that the dual gap configurations can offer high damping force and dynamic range, the current designs may not suitable for off-road vehicles as they don’t permit large stroke, apart from large dynamic force and dynamic range. It should be noted that off-road vehicles are normally subjected to harsh loading conditions (e.g. high frequencies and amplitudes) that require high damping force, and dynamic range, together with large piston stroke. Furthermore, the current designs do not meet the reasonably well compact design requirement to be easily integrated into such applications.

In this paper, a compact bypass annular-radial MR damper adapted for off-road tracked vehicles has been developed, modeled, and optimally designed. The mathematical formulation was established based on the MR fluid Bingham plastic model. Firstly, the performance of the valve’s magnetic circuit has been evaluated analytically by calculating the magnetic flux density in the MR fluid active region. The analytical results were then verified using a magnetic finite element method. The MR valve’s geometrical dimensions have been subsequently optimized to achieve a high dynamic range. The GA and SQP algorithms in a successive manner were utilized to obtain the global optimum solution. The developed optimally designed MR damper has been finally fabricated, assembled, and experimentally tested. The validation of the analytical model and high dynamic range of the proposed MR damper were further provided.

## 2. Principal and configuration of the proposed MR damper

### 2.1. Principal of the design

**Figure 1.**
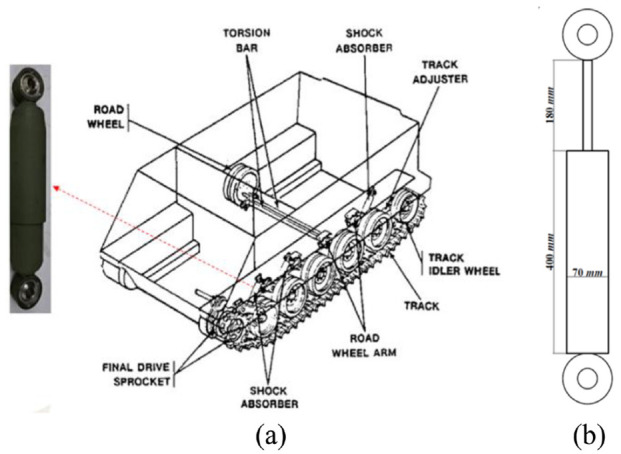
M113 off-road vehicle suspension system: (a) M113 tracked vehicle undercarriage components (Sutey, 1992) and (b) shock absorber’s dimensions.

The proposed MR damper was designed to be adapted for off-road tracked vehicles operating in harsh conditions for defense applications. Even at low excitation frequencies and modest displacement amplitudes, these vehicles require substantial damping force, high dynamic range, and a large stroke shock absorber. The existing hydraulic shock absorber of M113 tracked vehicles, shown in Figure 1, limits the vehicle mobility, speed, maneuverability, and adaptability for various operating conditions and excitations (Rosenfeld and Wereley, 2004). This conventional damper has a stroke of 180 mm and it can generate a constant damping force equal to 467 N at low piston rod speed of 12.5 mm/s (Dhir, 1993). As a result, the proposed MR damper in this study was developed in order to match the size and capacity of the M113 tracked vehicle’ shock absorber that has the dimension show in Figure 1(b). The aim is to replace the passive shock absorber with an MR damper having large dynamic range and stroke to reduce the vehicle vibration under a wide range of loading circumstances. Therefore, the vehicle dynamic and maneuverability will be improved, as suggested in other studies (Abdalaziz et al., 2022; Ata, 2014; Deo and Suh, 2005; Els et al., 2007; Long and Comito, 1979).

### 2.2. Configuration of the MR damper

**Figure 2.**
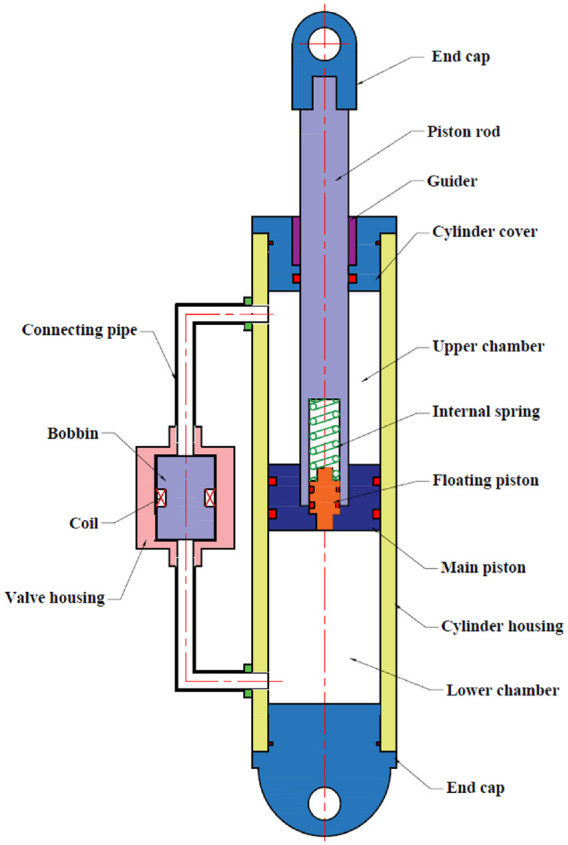
Schematic of the proposed MR damper.

The proposed MR damper, shown in Figure 2, consists of a main piston, piston rod, and floating piston connected with a mechanical spring inside a cylindrical housing and an external MR fluid bypass valve. The cylindrical housing is composed of two chambers, upper and lower chambers, which are filled with MR fluid. The piston rod on the side that connected to the piston has an internal hole which accommodates an internal spring connected to a floating piston. Instead of using gas chamber commonly used in conventional MR damper, the integrated mechanical internal spring connected to the floating piston alternatively functions as an accumulator. This unit permits pressurizing approximately 1000 ml of MR fluid to prevent cavitation on the low pressure induced by piston motion as well as compensate the change in fluid volume due to the movement of piston rod. In addition, integrating the accumulator inside the piston rod provides a more compact design and also allows increasing the damper stroke in a given specific volume. The other end of piston rod is sliding through sealed guider. The external MR bypass valve consists of an outer shell, a bobbin with an imbedded electromagnetic coil, and a spacer allowing the flow of MR fluid in both annular and radial gaps. The proposed MR damper has been optimally designed to be compact while providing variable damping force and high dynamic range. It is worth noting that the assembly and maintenance of the proposed damper as well as MR valve are quite simple due to the absence of the gas chamber and internal MR valve. Furthermore, considering the space requirement in the radial direction within M113’s suspension system, the MR valve is designed to be miniaturized, thereby occupying minimum radial space, and thus fitting the suspension area. Likewise, the hydraulic housing, connecting the upper and lower chambers, can be slightly shorter/longer depending on the type of application. This permits modification of the MR valve’s position with respect to the damper to properly fit the space.

## 3. Mathematical formulation of the MR damper

**Figure 3.**
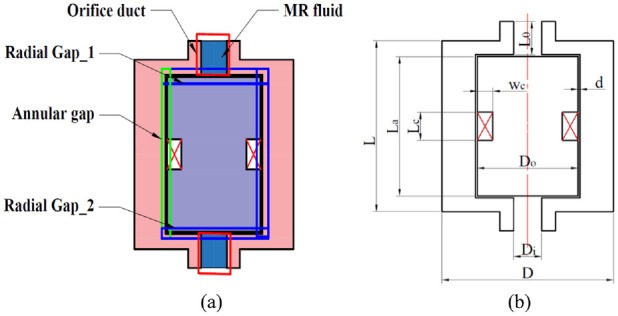
MR fluid bypass valve configuration: (a) MR valve gaps and orifices layout and (b) MR valve main dimensions.

The proposed MR bypass valve is used to regulate the damping force via activation of MR fluids passing through both annular and radial gaps as shown in Figure 3(a). By assuming Bingham plastic characteristics for MR fluid and neglecting the unsteady MR fluid effect, friction force, inertia effect, and compressibility of the MR fluid, the generated total damper force can be written as:

(1) $$F_{d}=F_{s}+F_{\eta }+F_{\tau }$$

The total damper force is a combination of the spring force ( $F_{s}$ ), viscous force ( $F_{\eta }$ ), and controllable yield force ( $F_{\tau }$ ). $F_{\eta }$ and $F_{\tau }$ are, respectively, related to the pressure drop due to viscous resistance ( $\Delta P_{\eta }$ ) and yield shear stress ( $\Delta P_{\tau \;}$ ) generated due to the application of the magnetic field to MR fluid through gaps. Hence equation (1) may be written as:

(2) $$F_{d}=F_{s}+\left(\Delta P_{\eta }+\Delta P_{\tau \;}\right)\left(A_{p}-A_{r}\right)$$

where $A_{p}$ and $A_{r}$ respectively, are the cross-sectional area of the piston and piston rod.

Based on the Bingham-plastic model, the mathematical expression describing the pressure drop in the annular gap $(\Delta P_{a}$ ) and the pressure drop in the radial gap $,(\;\Delta P_{r}$ ) due to viscous and fluid shear stress and the pressure drop in the orifice duct ( $\Delta P_{o}$ ), due to viscous damping can be formulated as:

(3) $$\Delta P_{a}=\Delta P_{\eta a}+\Delta P_{\tau a}=\frac{12\eta L_{a}Q}{\pi D_{m}d^{3}}+\frac{c_{a}(L_{a}-L_{c})}{d}\tau_{\mathrm{ya}}\left(B\right)$$

(4) $$\Delta P_{r}=\Delta P_{\eta r}+\Delta P_{\tau r}=\frac{6\eta Q}{\pi d^{3}}\ln\left(\frac{D_{o}}{D_{i}}\right)+\frac{c_{r}\tau_{\mathrm{yr}}(B)}{2d}\left(D_{o}-D_{i}\right)$$

(5) $$\Delta P_{o}=\frac{128\;\eta L_{o}Q}{\pi {D_{i}}^{4}}$$

where

(6) $$D_{m}=(D_{o}+d)$$

where in the above equations, $\Delta P_{\eta a}$ , $\Delta P_{\tau a}$ , $\Delta P_{\eta r}$ , and $\Delta P_{\tau r}$ are the pressure drop due to viscous and shear stress in the annular and radial gaps respectively. *d* is the flow channel annular or radial gap size, *η* is the fluid base viscosity (the field-independent plastic viscosity), Q is the volume flow rate, $\;L_{o}$ is the orifice duct length, *L_a_* is the length of the annular flow duct, *L_c_* is the MR valve coil length, *D_m_* is the annular channel diameter, *D_i_* and *D_o_* represent the radial duct length from input to the output of the flow channel, *τ* (*B*) is the field-dependent yield stress of the MR fluid, *B* is the magnetic flux density. $c_{a}$ , and $c_{r}$ are the flow velocity profile function coefficient of the annular (radial) gap.

Since the MR fluid bypass valve consists of two orifices, two radial gaps, and one annular gap, then the total pressure drop within the MR valve ( $\Delta P_{v})$ can be calculated as:

(7) $$\Delta P_{v}=(\Delta P_{\eta a}+2\Delta P_{\eta r}+2\Delta P_{o})+(\Delta P_{\tau a}+2\Delta P_{\tau r})$$

Thus, by substituting equations (3)–(5) into equation (7), the total pressure drop can be expressed as:

(8) $$\begin{array}{c} \Delta P_{v}=\left[\frac{12\eta L_{a}Q}{\pi D_{m}d^{3}}+\frac{12\eta Q}{\pi d^{3}}\ln\left(\frac{D_{o}}{D_{i}}\right)+\frac{256\;\eta L_{o}Q}{\pi {D_{i}}^{4}}\right]\\ \;+\left[\frac{c_{a}\left(L_{a}-L_{c}\right)\tau_{\mathrm{ya}}}{d}+\frac{c_{r}\left(D_{o}-D_{i}\right)\tau_{\mathrm{yr}}}{d}\right] \end{array}$$

It is noted that the Q in equation (3) can be obtained as:

(9) $$Q=(A_{p}-A_{r}){\overset{\cdot }{x}}_{p}$$

where ${\overset{\cdot }{x}}_{p}$ is the piston velocity, $c_{a(r)}$ , the flow velocity profile function coefficient of the annular (radial) gap which can be evaluated with respect to the ratio between pressure drop due to field-dependent shear stress to the viscous pressure drop. $c_{a(r)}$ ranges from a minimum value of 2.07 to a maximum value of 3.07 based on equation (10) or it can be calculated approximately using equation (11), which is described in Nguyen et al. (2008) as:

(10) $$c_{a\left(r\right)}=\left\{\begin{array}{c} 2.07,\;\frac{\Delta P_{\tau a\left(r\right)}}{\Delta P_{\eta a\left(r\right)}}<1\;\\ 3.07,\;\frac{\Delta P_{\tau a\left(r\right)}}{\Delta P_{\eta a\left(r\right)}}>1\; \end{array}\right\}$$

(11) $$c_{a(r)}=2.07+\frac{12Q\eta }{12Q\eta +1.6\pi D_{m}d^{2}\tau_{\mathrm{ya}(r)}}$$

Hence, the total damping force presented in equation (1) can also be described in the following form:

(12) $$F_{d}=F_{s}+c_{\mathrm{vis}}{\overset{\cdot }{x}}_{p}+F_{\tau }\mathrm{sgn}\left({\overset{\cdot }{x}}_{p}\right)$$

where $c_{\mathrm{vis}}$ is the passive viscous coefficient. The spring force can be calculated as:

(13) $$\;F_{s}=k\left(\frac{A_{r}-A_{f}}{A_{f}}\right)x_{p}$$

where k is the stiffness coefficient of the inner spring that is set at 120 kN/m, and $A_{f}$ is the floating piston area. $D_{f\;}$ is floating piston diameter, which is equals to 19.05 mm. Using equations (2) and (7)−(9), the $c_{\mathrm{vis}}$ and $F_{\tau },$ can be obtained as:

(14) $$C_{\mathrm{vis}}=\left[\frac{12\eta L_{a}}{\pi D_{m}d^{3}}+\frac{12\eta }{\pi d^{3}}\ln\left(\frac{D_{o}}{D_{i}}\right)+\frac{256\eta L_{o}}{\pi {D_{i}}^{4}}\right](A_{p}-A_{r}{)}^{2}$$

and

(15) $$F_{\tau }=\left[\frac{c_{a}\left(L_{a}-L_{c}\right)\tau_{\mathrm{ya}}}{d}+\frac{c_{r}\left(D_{o}-D_{i}\right)\tau_{\mathrm{yr}}}{d}\right]\left(A_{p}-A_{r}\right)$$

The equivalent field-dependent viscous damping coefficient may also be formulated as:

(16) $$C_{\mathrm{eq}\;}=\frac{\;F_{s}+C_{\mathrm{vis}}{\overset{\cdot }{x}}_{p}+F_{\tau }\mathrm{sgn}\left({\overset{\cdot }{x}}_{p}\right)}{{\overset{\cdot }{x}}_{p}}$$

It should be noted that providing a wide control range of the MR damper force depends on the dynamic range of the damper. It is defined as the ratio of the peak damping force under a maximum current input to the damping force generated under zero current input. Alternatively, it is the ratio of the total force to the viscous force and can be mathematically described as:

(17) $$\lambda_{d}=\frac{F_{\mathrm{on}}}{F_{\mathrm{off}}}=\frac{\;F_{s}+F_{\eta }+F_{\tau }}{\;F_{s}+F_{\eta }}=\frac{\;F_{s}+C_{\mathrm{vis}}{\overset{\cdot }{x}}_{p}+F_{\tau }\mathrm{sgn}({\overset{\cdot }{x}}_{p})}{\;F_{s}+C_{\mathrm{vis}}{\overset{\cdot }{x}}_{p}}$$

The MR fluid (MRF-132DG) for the proposed MR damper is purchased from the Lord corporation (Lord Corporation, 2011). This MR fluids has a dark gray appearance with viscosity of 0.112 Pa s and average density of 3.05 g/cm^3^. Based on the available experimental testing of the MRF-132DG, the field-dependent shear yield stress ( $\tau_{\mathrm{ya}(r)}\left(B\right)$ ) in the annular and radial gaps $\tau_{\mathrm{ya}(r)}$ can be estimated as (Hemmatian et al., 2020):

(18) $$\tau_{\mathrm{ya}(r)}\left(B\right)=2.7B_{a\left(r\right)}^{3}-26.467B_{a\left(r\right)}^{2}+66.643B_{a\left(r\right)}-2.4295$$

By evaluating the magnetic flux density in the annual and radial gaps at different applied currents, the $\tau_{\mathrm{ya}(r)}\left(B\right)$ can be calculated using equation (18). Subsequently by having the geometrical parameters of the MR valve, damper, and piston velocity, the generated damping force can be subsequently determined using equations (12)–(15). In this study, the initial geometrical parameters of the MR damper (valve geometrical parameters are shown in Figure 3(b)) are chosen based on the capacity, size, and damping force of the conventional shock absorber of M113 tracked vehicle, mentioned in section 1. These geometrical parameters are summarized in Table 1.

**Table 1.** The proposed MR damper initial parameters.

Parameter	Symbol	Value (mm)
Duct gap	d	0.9
Coil width	$w_{c}$	7
Piston diameter	$D_{p}$	55
Piston rod diameter	$D_{r}$	30
Radial duct outer diameter	$D_{o}$	32.1
Radial duct inner diameter	$D_{i}$	10
MR valve whole diameter	D	90
Annular duct length	$L_{a}$	50
Coil length	$L_{c}$	10
MR valve height	L	67.8

### 3.1. Analytical magnetic circuit analysis of MR valve

**Figure 4.**
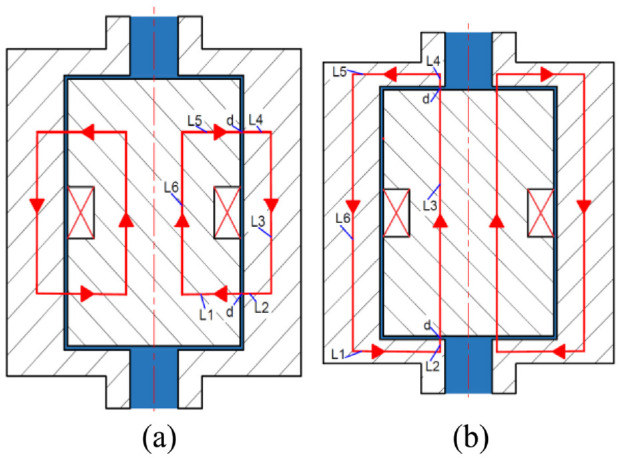
MR valve magnetic circuit: (a) annular gap circuit and (b) radial gap circuit.

In this study, the magnetic circuit analysis has been conducted to evaluate the magnetic flux density in the MR fluid within active regions of the MR valve for different coil currents. First an approximate analytical approach based on the Ampere’s law has been formulated and the results have then been compared with those found using numerical approach based on the open-source magneto-static finite element method magnetic (FEMM) software. The equivalent magnetic circuit path for the proposed MR fluid bypass valve with annular and radial gaps is shown in Figure 4. Using the Ampere’s law, the relation between the magnetic field intensity and the applied current through the MR valve coil can be described as:

(19) $$N_{c}I=\sum H_{j}L_{j}$$

where $N_{c}$ is the number coil turns in the electromagnet, I is the applied current, $H_{j}$ is the magnetic field intensity for j th circuit links (or elements), and $L_{j}$ is the effective length of this link. The mathematical expression for the magnetic flux conservation through the circuit as well as the relation between the magnetic flux density and the magnetic flux intensity can be written as:

(20) $$Ø=B_{j}A_{j}$$

(21) $$B_{j}=\mu_{o}\mu_{\mathrm{rj}}H_{j}=\mu_{j}H_{j}$$

where Ø is the magnetic flux passing within the circuit and $A_{j}$ is the effective cross-sectional area at the midpoint of j th link, $B_{j}$ is the magnetic flux density of the j th link. $\mu_{o}$ is the magnetic permeability of free space ( $\mu_{o}$ = 4π χ 10^–7^ H/m) and $\mu_{\mathrm{rj}}$ is the relative magnetic permeability of the material, which has a considerable effect in the evaluation of the magnetic flux density. It is noted that at low applied magnetic field the relationship between B and H is linear, however as the applied magnetic field intensity increases, the relationship becomes nonlinear due to the saturation of the magnetic induction at high magnetic fields. This is mainly due to the dependency of $\mu_{j}$ to the applied magnetic field, as the magnetic field intensity increases. In analytical magnetic circuit analysis, it is assumed that the applied magnetic field intensity is well below the saturation so that the linear assumption is valid.

The magnetic circuit within the designed MR valve can travel within both the annular and radial gaps as shown, respectively, in Figure 4(a) and (b). The magnetic circuit of the single-coil MR bypass valve configuration for both gaps is divided into total eight links. Six of these links represent the metallic core links for bobbin and the rest of two links represent the MR fluid within two gaps. Hence, equations (19) and (20) can be formulated for the annular and radial gaps as:

(22) $$N_{c}I=\sum_{j=1}^{6}L_{j}H_{j}+2dH_{f}$$

(23) $$Ø=B_{f}A_{f}=B_{j}A_{j}$$

where $H_{f}$ and $B_{f}$ are magnetic flux intensity and density through the MR fluid gap respectively. By substituting equation (21) into equation (22), the equation (22) can be further simplified as:

(24) $$N_{c}I=\sum_{j=1}^{6}\frac{B_{j}L_{j}}{\mu_{j}}+2d\frac{B_{f}}{\mu_{f}}$$

It is noted that $\mu_{j}$ is the permeability of the valve core and housing material and $\mu_{f}$ is the permeability of the MR fluid. Substituting the equation (23) into equation (24) yields:

(25) $$N_{c}I=\sum_{j=1}^{6}\frac{B_{f}A_{f}L_{j}}{\mu_{j}A_{j}}+2d\frac{B_{f}}{\mu_{f}}$$

or

(26) $$N_{c}I=B_{f}\left[A_{f}\sum_{j=1}^{6}\left(\frac{L_{j}}{\mu_{j}A_{j}}\right)+2\frac{d}{\mu_{f}}\right]$$

where

(27) $$A_{f}=\left\{\begin{array}{cc} \pi D_{m}d,\; & \;\mathrm{for}\;\mathrm{annular}\;\mathrm{duct}\\ \frac{\pi }{4}\left(D_{o}^{2}-D_{i}^{2}\right)\;, & \mathrm{for}\;\mathrm{radial}\;\mathrm{gab} \end{array}\right\}$$

Now using equation (26), the induced magnetic flux density in the MR fluid within active regions can be estimated as:

(28) $$B_{f}=\frac{N_{c}I}{\frac{2d}{\mu_{f}}+A_{f}S_{j}}$$

where $S_{j}$ is the total reluctance of the bobbin’s links, which can be described as:

(29) $$S_{j}=\;\sum_{1}^{6}\frac{L_{j}}{\mu_{j}A_{j}}$$

The magnetic circuit parameters for both annular and radial gaps of the MR valve have been formulated and are summarized in Tables 2 and 3, respectively. The number of coil turns in electromagnet can also be estimated using relation reported in Shamieh and Sedaghati (2017) as:

(30) $$N_{c}=\frac{\;2w_{c}L_{c}}{1.68d_{w}^{2}}$$

**Table 2.** Magnetic circuit parameters of the MR fluid bypass valve (annular gap).

Link no	Length $L_{j}$	Area $A_{j}$	Reluctance $S_{j}$
1	$L_{1}=\frac{\left(D_{o}+2w_{c}\right)}{4}$	$A_{1}=2\pi \left(\frac{L_{a}-L_{c}}{2}\right)\left(\frac{3D_{o}+2w_{c}}{8}\right)$	$S_{1}=\frac{L_{1}}{\mu_{s}A_{1}}$
2	$L_{2}=\frac{\left(D-D_{o}-2d\right)}{4}$	$A_{2}=2\pi \left(\frac{D-D_{o}-2d}{2}\right)\left(\frac{D+3D_{o}+6d}{8}\right)$	$S_{2}=\frac{L_{2}}{\mu_{s}A_{2}}$
3	$L_{3}=\frac{L_{a}+L_{c}}{2}$	$A_{3}=\pi \left[\frac{D^{2}}{4}-{\left(\frac{D+D_{o}+2d}{4}\right)}^{2}\right]$	$S_{3}=\frac{L_{3}}{\mu_{s}A_{3}}$
4	$L_{4}=\frac{\left(D-D_{o}-2d\right)}{4}$	$A_{4}=2\pi \left(\frac{D-D_{o}-2d}{2}\right)\left(\frac{D+3D_{o}+6d}{8}\right)$	$S_{4}=\frac{L_{4}}{\mu_{s}A_{4}}$
5	$L_{5}=\frac{\left(D_{o}+2w_{c}\right)}{4}$	$A_{5}=2\pi \left(\frac{L_{a}-L_{c}}{2}\right)\left(\frac{3D_{o}+2w_{c}}{8}\right)$	$S_{5}=\frac{L_{5}}{\mu_{s}A_{5}}$
6	$L_{6}=\frac{L_{a}+L_{c}}{2}$	$A_{6}=\pi {\left(\frac{D_{o}-2w_{c}}{2}\right)}^{2}$	$S_{6}=\frac{L_{6}}{\mu_{s}A_{6}}$

**Table 3.** Magnetic circuit parameters of the MR fluid bypass valve (radial gap).

Link no	Length $L_{j}$	Area $A_{j}$	Reluctance $S_{j}$
1	$L_{1}=(\frac{D+2d+2w_{c}}{4})$	$A_{1}=2\pi \left(\frac{L-L_{c}}{2}\right)\left(\frac{2D+D_{o}+2w_{c}}{8}\right)$	$S_{1}=\frac{L_{1}}{\mu_{s}A_{1}}$
2	$L_{2}=\frac{L+L_{a}+2d}{2}$	$A_{2}=\pi \left[\frac{D^{2}}{4}-{\left(\frac{D+D_{o}+2d}{4}\right)}^{2}\right]$	$S_{2}=\frac{L_{2}}{\mu_{s}A_{2}}$
3	$L_{3}=(\frac{D+2d+2w_{c}}{4})$	$A_{3}=2\pi \left(\frac{L-L_{c}}{2}\right)\left(\frac{2D+D_{o}+2w_{c}}{8}\right)$	$S_{3}=\frac{L_{3}}{\mu_{s}A_{3}}$
4	$L_{4}=\frac{L-L_{a}-2d}{4}$	$A_{4}=\frac{\pi }{4}\left[D^{2}-D_{i}^{2}\right]$	$S_{4}=\frac{L_{4}}{\mu_{s}A_{4}}$
5	$L_{5}=L_{a}$	$A_{5}=\pi {\left(\frac{D_{o}-2w_{c}}{2}\right)}^{2}$	$S_{5}=\frac{L_{5}}{\mu_{s}A_{5}}$
6	$L_{6}=\frac{L-L_{a}-2d}{4}$	$A_{6}=\frac{\pi }{4}\left[D^{2}-D_{i}^{2}\right]$	$S_{6}=\frac{L_{6}}{\mu_{s}A_{6}}$

### 3.2. Magneto-static finite element analysis

**Figure 5.**
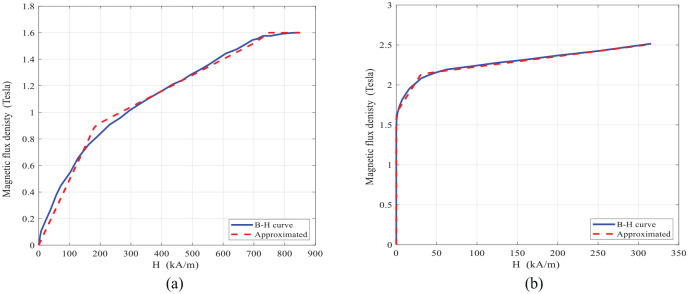
The B-H curve of MR fluid and bypass valve material: (a) B-H curve MRF-132 DG (Lord Corporation, 2011) and (b) B-H curve steel 1006 (Salloom and Samad, 2011).

Finite element analysis of the MR fluid bypass valve has also been conducted using an open source Finite Element Method Magnetics (FEMM) software (Baltzis, 2010). FEMM can be effectively used to accurately predict the intensity and distribution of magnetic flux in the magnetic circuits (DC or low frequency applied currents) on two-dimensional axisymmetric domain. The FE model was then developed using FEMM using geometrical and material specifications corresponding to the designed MR fluid bypass valve. The B–H curves of the MR fluid (MRF-132DG) and the magnetic material of the valve core and housing (AISI 1006) are shown in Figure 5(a) and (b), respectively.

Using the curve-fitting, the following piecewise linear relations can be used in FEMM to describe the B-H behavior of MR fluid as well as magnetic core material and housing as:

(31) $$B_{f}=\left\{\begin{array}{cc} 0.00049\;H, & 0\leq H\leq 200\\ 0.6814+0.0012H, & \;200\leq H\leq 700\\ 1.6\;, & H\;>700 \end{array}\right\}$$

(32) $$B_{s}=\left\{\begin{array}{cc} 1.5, & H\leq 1\\ 1.6467+0.0159H, & 1\leq H\leq 30\\ 2.0969+0.0013H, & \;30\leq H\leq 300\\ 2.5, & H\;>300 \end{array}\right\}$$

where $B_{f}$ is the MR fluid magnetic flux density and $B_{s}$ is the magnetic flux density of the valve’s material (AISI 1006).

The MR valve coil wire is chosen to be Gauge 22 AWG copper wire with a resistance per unit length of 52.96 mΩ/m. and maximum applied current of 7A . As mentioned before the field-dependent yield strength of the employed MR fluid, MRF-132DG, under varied applied magnetic flux density can be obtained using equation (18). The average magnetic flux density in the MR fluid active regions for annular and radial gaps has been evaluated by averaging the induced magnetic flux density along the length of the annular and radial gaps using the following relation:

(33) $$B_{\mathrm{av}}=\frac{1}{l}\int_{0}^{l}B\left(s\right)\;\mathrm{ds}$$

## 4. Design optimization formulation of the MR damper

The dynamic range of the MR fluid bypass valve is an important performance index to be considered in design optimization of MR dampers. Increasing the dynamic range of the MR damper will greatly enhance its capability to attenuate vibration under wide range of frequencies. The objective of the proposed design optimization is to formulate a multidisciplinary problem in order to identify the geometrical and magnetic circuits parameters, which maximize the dynamic range of the proposed MR damper under given volume, magnetic, and damping force constraints.

**Figure 6.**
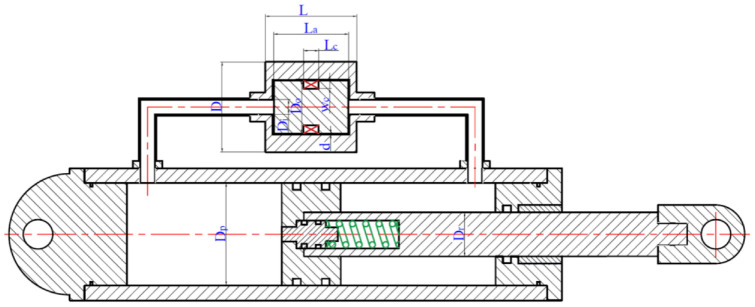
By-pass MR damper with main identified design parameters.

The main design variables that represent the geometrical parameters of the MR damper and MR fluid bypass valve and its magnetic circuit are $d,w_{c},D_{p},D_{r},D_{o},D_{i},D,L_{a},L_{c}$ , and L , as shown in Figure 6. For the sake of clarity, the description of the design variables together with their lower and upper bounds are summarized in Table 4. The MR fluid properties and the magnetic circuit parameters are provided in Table 5. The piston velocity is considered to be 12.5 mm/s.

**Table 4.** Main design parameters and their assigned lower and bounds.

No	Description	Variables	Bound (mm)
1	Duct gap	$x_{1}=d$	0.5–2
2	Coil width	$x_{2}=w_{c}$	5–20
3	Piston diameter	$x_{3}=D_{p}$	40–70
4	Piston rod diameter	$x_{4}=D_{r}$	20–50
5	Radial gap outer diameter	$x_{5}=D_{o}$	10–80
6	Radial duct inner diameter	$x_{6}=D_{i}$	5–30
7	MR valve whole diameter	$x_{7}=D$	50–90
8	Annular gap length	$x_{8}=L_{a}$	20–70
9	Coil length	$x_{9}=L_{c}$	5–25
10	MR valve height	$x_{10}=L$	50–80

**Table 5.** MR fluid and valve material properties.

Parameter	Symbol	Value	Unit
MR fluid density	ρ	3050	kg/m^3^
MR fluid viscosity	η	0.112	Pa s
MR fluid relative permeability	$\mu_{f}$	5	
Steel (1006) relative permeability	$\mu_{j}$	1404	
Permeability of free space	$\mu_{o}$	$4\pi \times 10^{-7}$	TmA^−1^
Copper wire (gage AWG22) diameter	$d_{w}$	0.7131	mm

Constraints imposed on the MR fluid bypass valve have been categorized into geometrical, and physical (e.g. magnetic circuit) constraints. Side constraints are considered for all design parameters (see Table 4), so that design optimization can be conducted within a given design space and volume ( $V_{\mathrm{mr}})$ , thus allowing a compact design of the MR fluid bypass valve. Also, the gap size has been identified as an important parameter to be optimized as it significantly affects the dynamic performance of the MR valves (Hu et al., 2017, 2018). Wider gaps generally reduce the viscous and controllable yield damping forces, while a smaller gap increases the pressure drop but it may also cause valve blockage. Practically, the gap size ranges between 0.5 and 2 mm (Ai et al., 2006; Fatah et al., 2015; Hu et al., 2018), depending on the design requirements. Moreover, the induced magnetic flux density in the MR fluid’s active regions should be lower than the saturation limit of the MR fluid. This avoids temperature rise in the coil, thereby allowing valve to operate continuously over a longer period of time. Also, the magnetic flux saturation of the MR valve’s core material should not occur before that of MR fluid. Here a factor ( γ ) is defined as the ratio of the magnetic flux in the MR valve bobbin to that in the MR fluid. To guard against saturation limits and also provide compact design the factor γ has been limited to the range of 1.02 and 1.07 (Saleh et al., 2019). It is noted that the saturation limit of the MR fluid is approximately 1.6 Tesla, as shown in Figure 5(a) and the core (steel AISI 1006) is saturated approximately at 2.5 Tesla.

The off-state damping force should not be less than 467 N based on the specification of the vehicle conventional shock absorber in Figure 1. The minimum yield damping force of the MR damper is also considered to be at least 950 N to assure dynamic range of greater than 2. Considering the above-mentioned design requirements, the design optimization problem can be formally formulated as:

Find $X=\left[d,w_{c},D_{p},D_{r},D_{o},D_{i},D,L_{a},L_{c},L\right]$ , a design variable vector of dimension 10, to minimize

(34) $$f(X)=\frac{\;F_{s}+C_{\mathrm{vis}}{\overset{\cdot }{x}}_{p}}{\;F_{s}+C_{\mathrm{vis}}{\overset{\cdot }{x}}_{p}+F_{\;\tau }\mathrm{sgn}({\overset{\cdot }{x}}_{p})\;}=\frac{F_{\mathrm{off}}}{F_{\mathrm{on}}}=\frac{1}{\lambda_{d}}$$

Subject to

(35) $$\left\{\begin{array}{c} -\frac{F_{\mathrm{off}}}{467\;}+1\leq 0\;N\;\\ -\frac{F_{\tau }}{950}+1\leq 0\;N\\ \frac{V_{\mathrm{mr}}}{4.31\times 10^{5}}-1\leq 0\;mm^{3}\\ -\gamma +1.02\leq 0\\ \gamma -1.07\leq 0\\ 0.5\leq x_{1}\leq 2\;\mathrm{mm}\\ \;5\;\leq x_{2}\leq 20\;\mathrm{mm}\\ \;40\leq x_{3}\leq 70\;\mathrm{mm}\\ \;20\;\leq x_{4}\leq 50\;\mathrm{mm}\\ \;10\;\leq x_{5}\leq 80\;\mathrm{mm}\\ \;5\;\leq x_{6}\leq 30\;\mathrm{mm}\\ \;50\leq x_{7}\leq 90\;\mathrm{mm}\\ \;20\;\leq x_{8}\leq 70\;\mathrm{mm}\\ \;5\;\leq x_{9}\leq 25\;\mathrm{mm}\\ \;50\leq x_{10}\leq 80\;\mathrm{mm} \end{array}\right\}$$

The formulated design optimization problem was successfully solved using the successive implementation of GA, which is a stochastic-based optimization technique, and the SQP algorithm, which is a powerful nonlinear gradient-based optimization technique, to accurately capture the global optimum solution. Due to its stochastic nature, the GA can provide near global optimum solution. The optimum solution from GA is then used as the initial point for the SQP algorithm to accurately catch the global optimum solution. Using this hybrid approach, same optimal solutions were obtained using different randomly generated initial population for the GA.

## 5. Results and discussion

The performance (e.g. damping force and dynamic range) of the initial design of the MR bypass valve (Table 1) are firstly evaluated and compared with those of optimally designed MR damper. The results of the magnetic circuit analyses including analytical and numerical approaches, have also been presented in this section.

### 5.1. Optimization results

**Figure 7.**
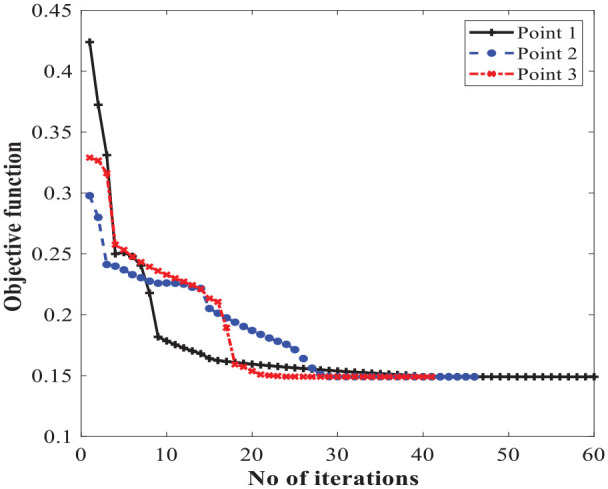
Iteration history of the optimized parameters.

As mentioned in section 4, the optimization problem was solved using a hybrid approach by consecutively combining GA and SQP algorithms to capture the true global optimum solution. Different initial populations for the GA are selected, which finally resulted in different optimum solutions near the global optimum. Table 6 provides the sample of optimum points obtained using GA. Subsequently, the optimal parameters obtained via GA, are deliberately chosen as the initial points for the SQP algorithm in order to guarantee a global optimum solution, which is summarized in Table 7. The iteration history of the SQP using the different initial points (Table 6) is graphically shown in Figure 7.

**Table 6.** The initially obtained optimal parameters via the Genetic Algorithm.

Iteration no	Design variables (mm)									
	$x_{1}$	$x_{2}$	$x_{3}$	$x_{4}$	$x_{5}$	$x_{6}$	$x_{7}$	$x_{8}$	$x_{9}$	$x_{10}$
1	2	6	50	40	50	10	50	60	24.6	80
2	0.9	12.3	70	21.2	25.6	5.1	51.3	35.5	13	50.5
3	0.5	4.4	47.2	34.9	26.8	5.3	80	30	10.3	80
4	0.6	15	65	30	49	6.5	90	26	13	60.3
5	0.7	7	69.9	30.6	45	10	70	30	11	61.6

**Table 7.** SQP global optimum solution (mm).

$x_{1}$	$x_{2}$	$x_{3}$	$x_{4}$	$x_{5}$	$x_{6}$	$x_{7}$	$x_{8}$	$x_{9}$	$x_{10}$
0.788	7	50	30	38.1	10	64.5	43.9	10.7	55.5

**Figure 8.**
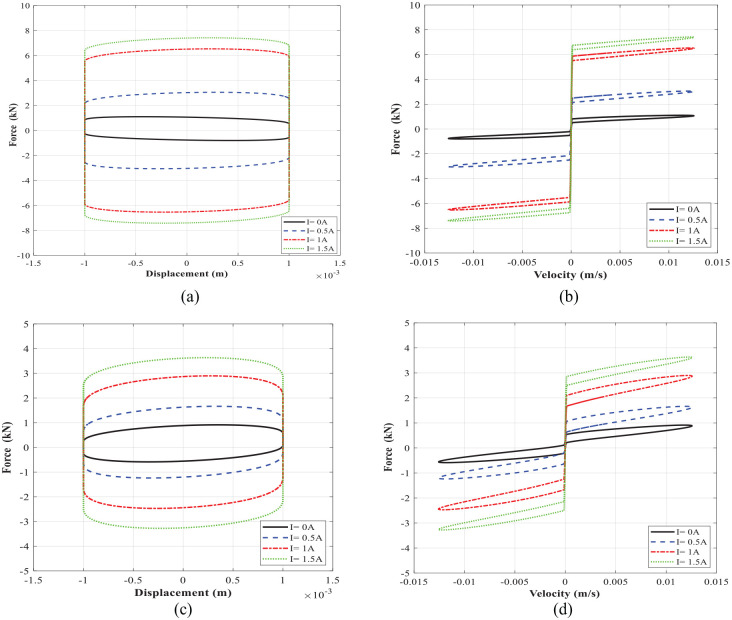
The comparison of force–displacement and force-velocity characteristics of the MR damper with the optimized MR valve (a and b) and with the initial design of MR valve (c and d) at loading frequency of 2 Hz and displacement of 1 mm.

Figure 8 compares the dynamic performance of the MR damper with the optimized (Figure 8(a) and (b)) and the initial (Figure 8(c) and (d)) designs of the MR valve in terms of force-displacement and force-velocity characteristics. The results are presented for the loading frequency of 2 Hz and displacement amplitude of 1 mm under varied levels of applied currents, ranging from 0 to 1.5 A. The optimized MR damper’s performance shows a remarkable improvement in terms of a relative increase in the enclosed area together with peak force, as shown in Figure 8(a) and (b) compared with the initial design of the MR damper’s performance, as shown in Figure 8(c) and (d). Such improvement can guarantee a relatively high dynamic range. For instance, the optimized damper can generate an on-state damping force of 7.41 kN and an off-state damping force of 1.1 kN, thereby yielding a large dynamic range of 6.7 under an applied current of 1.5 A. However, the initially designed damper based on Table 1 inputs revealed on-state and off-state damping forces of 3.63 and 0.91 kN, respectively, leading to a low dynamic range of 4. The optimized and initially designed geometrical parameters are compared and summarized in Table 8. As it can be realized the dynamic range of the optimized damper has been markedly improved by 67.5% as compared with the initially designed damper.

**Table 8.** Comparison of the initial and optimized parameters of the MR valve, along with its performance indices.

Parameter	Symbol	Initial design (mm)	Optimized design (mm)
Duct gap	d	0.9	0.788
Coil width	$w_{c}$	7	7
Piston diameter	$D_{p}$	55	50
Piston rod diameter	$D_{r}$	30	30
Radial duct outer diameter	$D_{o}$	32.1	38.1
Radial duct inner diameter	$D_{i}$	15	10
MR valve whole diameter	D	90	64.5
Annular duct length	$L_{a}$	50	43.9
Coil length	$L_{c}$	10	10.7
MR valve height	L	67.8	55.5
Performance Indices:			
Damping force (field-off), kN	$F_{\mathrm{off}}$	0.91	1.1
Max damping force (field-on), kN	$F_{\mathrm{on}}$	3.63	7.41
Dynamic range	$\lambda_{d}$	4	6.7

### 5.2. Magnetic circuit results

The solution of the magnetic circuit for the optimized MR valve was estimated analytically. The magnetic flux density *(B)* and magnetic field intensity *(H)* were approximately evaluated for both the annular and radial ducts under different excitation currents, as given in Table 9.

**Table 9.** Analytical results for annular and radial ducts magnetic circuit.

Current ( A )	Annular duct		Radial duct	
	Magnetic flux density (Tesla)	Magnetic field intensity (A/m)	Magnetic flux density (Tesla)	Magnetic field intensity (A/m)
0.10	0.054	$7.16\times 10^{3}$	0.0455	$6.03\times 10^{3}$
0.25	0.135	$1.79\times 10^{4}$	0.1138	$1.51\times 10^{4}$
0.50	0.270	$3.58\times 10^{4}$	0.2277	$3.02\times 10^{4}$
0.75	0.405	$5.37\times 10^{4}$	0.3420	$4.53\times 10^{4}$
1.00	0.540	$7.16\times 10^{4}$	0.4553	$6.04\times 10^{4}$
1.50	0.621	$8.24\times 10^{4}$	0.5236	$6.94\times 10^{4}$

**Figure 9.**
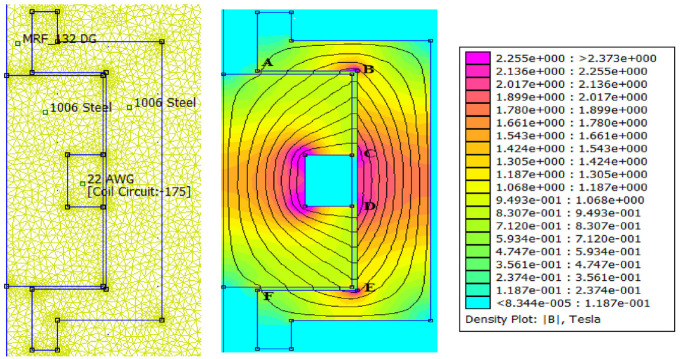
Magnetic flux distribution at excitation current 1.5 A.

**Figure 10.**
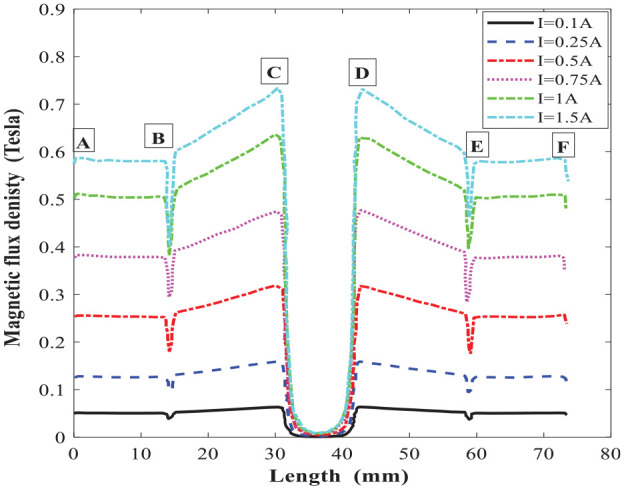
Magnetic flux density along the annular-radial path length under different applied current.

**Figure 11.**
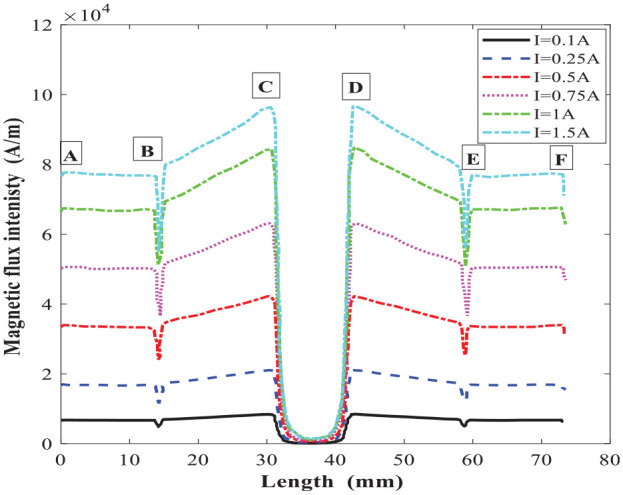
Magnetic flux intensity along the annular-radial path length under different applied current.

The magnetic circuit analytical results were then compared with simulation results based on the magnetic circuit finite element model developed in FEMM. A triangular mesh of 9327 elements and 4822 nodes were created with a precision of 1e-08. The meshing and the magnetic flux density distribution in the MR valve gaps and the MR valve linkages under excitation current 1.5 A are shown in Figure 9. The results for magnetic flux density and the distribution of the magnetic field intensity along the annular-radial path length (from A to F shown in Figure 9) are shown in Figures 10 and 11, respectively. The average results of the numerical solution for the magnetic circuit for different applied currents are summarized in Table 10.

**Table 10.** Average numerical results for annular and radial gaps magnetic circuit using FEMM.

Current ( A )	Annular duct		Radial duct	
	Magnetic flux density (Tesla)	Magnetic field intensity (A/m)	Magnetic flux density (Tesla)	Magnetic field intensity (A/m)
0.1	0.058	$7.5\times 10^{3}$	0.0504	$6.6\times 10^{3}$
0.25	0.146	$1.85\times 10^{4}$	0.125	$1.68\times 10^{4}$
0.5	0.293	$3.82\times 10^{4}$	0.253	$3.34\times 10^{4}$
0.75	0.426	$5.73\times 10^{4}$	0.378	$5\times 10^{4}$
1	0.584	$7.71\times 10^{4}$	0.504	$6.6\times 10^{4}$
1.5	0.667	$8.79\times 10^{4}$	0.58	$7.7\times 10^{4}$

**Figure 12.**
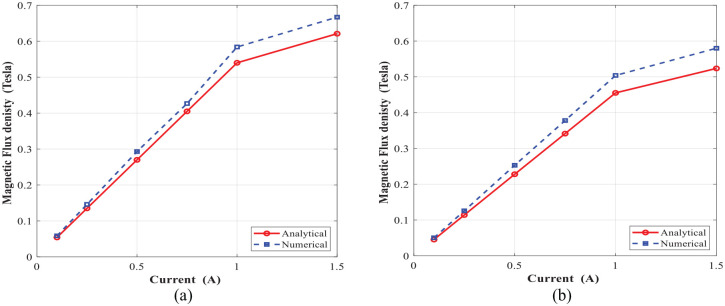
Magnetic flux density at different coil currents: (a) annular gap and (b) radial gap.

The comparison of the numerical and analytical results for the average magnetic flux density in the annular and radial gaps are shown in Figure 12(a) and (b), respectively. Results show a reasonably good agreement between the analytical and numerical results. Besides, results show that the relation between magnetic flux density and applied coil current is linear when coil current is below 1 A, irrespective of the gap type. Furthermore, the induced magnetic flux density is relatively higher within annular gap than that of radial gap, irrespective of coil current.

## 6. Experimental method

**Figure 13.**
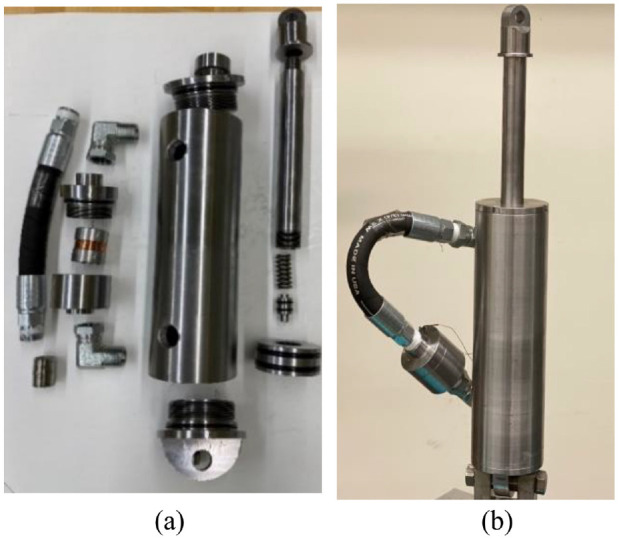
Fabricated MR damper: (a) MR damper components and (b) assembled MR damper.

Figure 13 shows the components and assembled configuration of the fabricated MR damper. These components have been fabricated with the proper tolerances and surface roughness. Alloy steel 4140 is selected as the material for cylinder housing and piston rod, while low carbon steel 1117 is selected for the main and floating pistons. The housing and bobbin of the MR fluid bypass valve are constructed of steel AISI 1006. The damper is filled with the commercial MR fluid 132-DG from Lord corporation.

Due to the limitation of the manufacturing tools and processing as well as cost-effectiveness, the manufactured MR damper slightly differs from the optimized MR damper. MR valve has a fluid gap of 1.2 mm instead of the optimal value of 0.778 mm while maintaining the dynamic range of at least 2 by enlarging the damper main piston diameter to 70 mm. For the MR coil wire that is chosen to be Gauge 24 AWG copper wire, the number of coil turns was increased to 215 turns to enhance the time response and overcome the short circuit problem that occurred. The core material is chosen to be steel AISI 1117 that has relative permeability of 1777 for overcoming the magnetic saturation due to increasing the number of turns. All the amendments are summarized in Table 11. The performance of the fabricated MR valve in terms of damping force, maximum damping force, and dynamic range, has been subsequently re-evaluated, via the mathematical formulations presented in Section 3. Table 11 also compares the performance indices of the fabricated MR valve with the theoretically optimized MR valve under peak piston velocity of 12.5 mm/s. As it can be seen from Table 11, the theoretical design optimization results show that the fabricated MR damper can provide the field-off damping force of 1.16 kN as well as field-on damping force and dynamic range of 5.43 kN and 4.68, respectively which are slightly lower than the optimized MR dampers.

**Table 11.** Comparison between the parameters and simulated performance of the optimized and fabricated MR bypass valve under applied current of 1.5 A.

Parameter	Symbol	Optimized MR damper (mm)	Fabricated MR damper (mm)
Duct gap	d	0.788	1.2
Coil width	$w_{c}$	7	7
Piston diameter	$D_{p}$	50	70
Piston rod diameter	$D_{r}$	30	30
Radial gap outer diameter	$D_{o}$	38.1	38.1
Radial gap inner diameter	$D_{i}$	10	10
MR valve whole diameter	D	64.5	64.5
Annular gap length	$L_{a}$	43.9	43.9
Coil length	$L_{c}$	10.7	10.7
MR valve height	L	55.5	60
Copper wire gage	$d_{w}$	AWG22	AWG24
Number of coils (turns)	*N_c_*	175	215
Performance indices			
Damping force (field-off), kN	$F_{\mathrm{off}}$	1.1	1.16
Max damping force (field-on), kN	$F_{\mathrm{on}}$	7.41	5.43
Dynamic range	$\lambda_{d}$	6.7	4.68

An experimental test has been designed to validate the simulation results for magnetic circuit of bypass MR valve and also to validate the predicted damping forces and dynamic range. Also, the fabricated MR damper has been experimentally characterized for evaluating its dynamic performance.

### 6.1. Experimental validation of the magnetic circuit model

**Figure 14.**
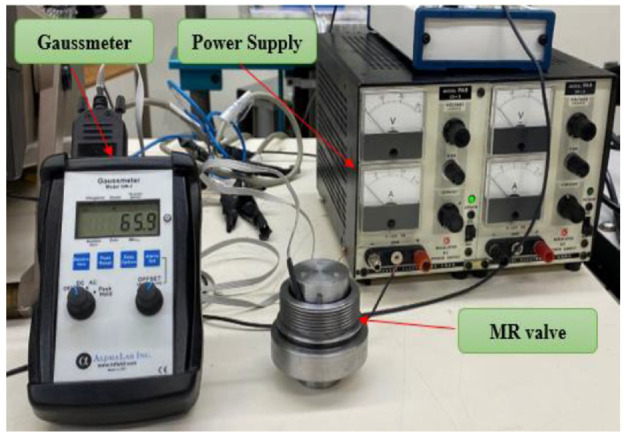
Measurement of the magnetic flux density along the MR damper’s annular gap.

The FE model developed for solving the magnetic circuit of the MR valve (along the annular MR fluid gap) was validated by measuring the steady-state magnetic flux density in the MR fluid gap. As it was not possible to directly measure the magnetic flux density in the MR fluid gaps regions in situ, the measurement has been conducted in the absence of MR fluid (in the air gap) by removing the valve from its enclosure. Then, the magnetic flux density was measured along the annular MR fluid gap. The results were compared to those obtained experimentally by locating Gauss meter at different locations along the annular gap. The setup of the experiment is shown in Figure 14. The MR valve coil was energized with different applied currents using a DC power supply (10 A, 100 V).

**Figure 15.**
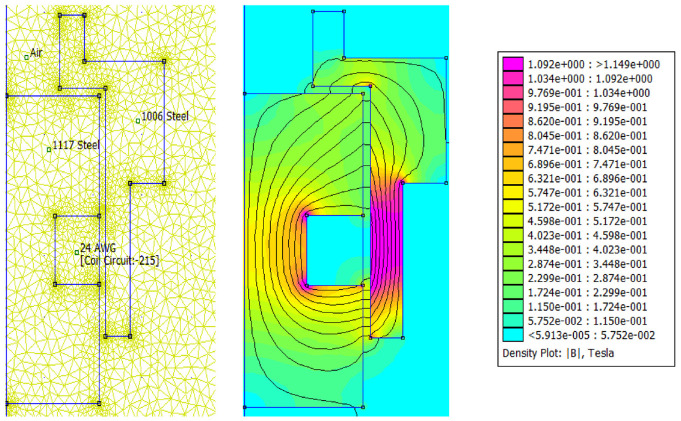
Magnetic flux distribution at excitation current 2 A.

**Figure 16.**
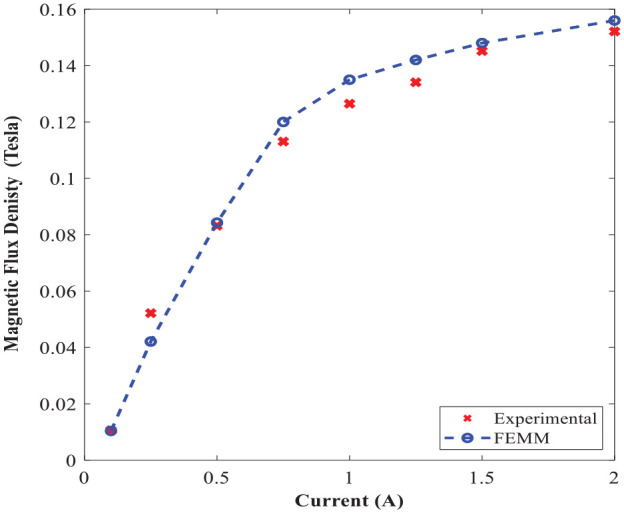
Comparison of the measured and FE estimation of the average magnetic flux density along the annular gap of the MR valve.

The magnetic flux density distribution using FEMM at the excitation current of 2 A is shown in Figure 15. The experimental results for the average magnetic flux density along the annular gap and their comparison with FE results under different applied currents are shown in Figure 16. Results are suggestive of a relatively well agreement between the experimentally and numerically obtained magnetic flux density. Besides, generally, the experimental results are slightly higher than that of FE simulation, which may be in part due to the experiment was conducted in the absence of MR fluid in the gap.

### 6.2. Force-displacement and force-velocity characteristics of the fabricated MR damper

**Figure 17.**
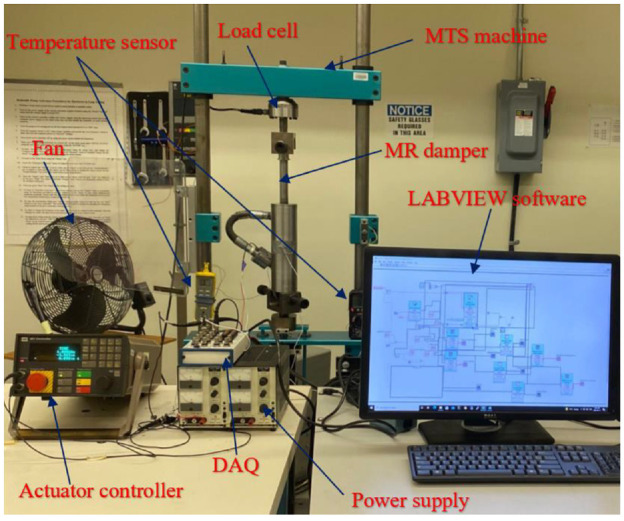
Test setup of the fabricated by-pass MR damper.

Experimental characterization of the fabricated MR damper was performed to verify the principle, advantages, and design optimization of the novel MR damper. The measurements were performed on a Material Testing System (MTS) machine, as shown in Figure 17. The MR damper was fixed between the upper and lower horizontal crossbeams of the loading frame of the MTS machine. The lower crossbeam contains a servo actuator that can apply sinusoidal excitations to the damper’s cylinder. A built-in Linear Voltage Displacement Transducer (LVDT) measures the displacement of the damper cylinder. The upper crossbeam contains a load cell to measure the damping force. The measurements are transmitted to a National Instruments Data Acquisition board (DAQ) then digitized and monitored on a PC using LabView software. The experimental measurements were performed for measuring force-displacement and force-velocity of the MR damper at low frequencies (1 and 2 Hz) and amplitudes of 1 mm respectively and under different excitation currents (0–2 A). The detailed experimental characterization is provided in Abdalaziz et al. (2022).

**Figure 18.**
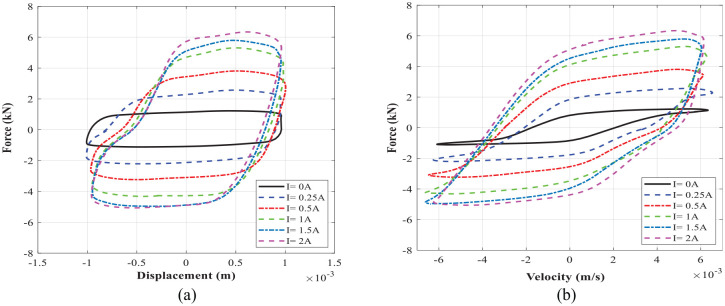
The measured data at excitation frequency of 1 Hz and displacement of 1 mm: (a) force-displacement and (b) force-velocity.

**Figure 19.**
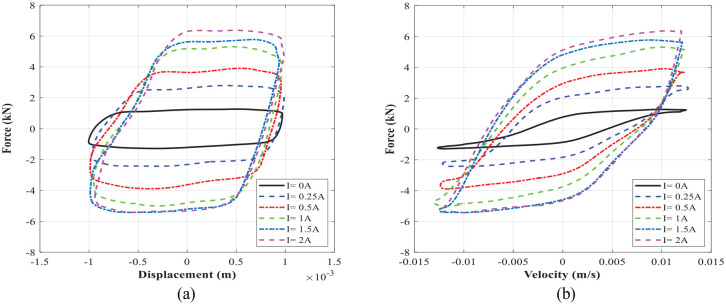
The measured data at excitation frequency of 2 Hz and displacement of 1 mm: (a) force-displacement and (b) force-velocity.

Experimental characterization of the MR damper is conducted by measuring the force-displacement and force velocity at each loading condition. Figure 18 shows force-displacement and force-velocity hysteresis behavior of MR damper under harmonic excitation with frequency of 1 Hz and loading amplitude of 1 mm, respectively. The presented results in Figure 18 were obtained at applied input current of 0–2 A. Figure 19 shows similar results for the excitation frequency of 2 Hz and loading amplitude of 1 mm. The results show that the area encircled by the force-displacement curve (representing the energy dissipation) substantially increases with increasing the applied current. The force-velocity curves also show that the damping force increases with increasing current as well as velocity. The developed MR damper can generate high peak damping forces and dynamic ranges. For instance, the damping force of the fabricated damper at the frequency of 2 Hz and displacement of 1 mm (peak velocity of 12.5 mm/s) ranges between 1.31and 5.9 kN from off-state to on state (current 1.5 A), yielding a large dynamic range of nearly 4.5 which are slightly lower than simulation results presented in Table 11. It is, however, noted that under an applied current of 2 A, the damping force and dynamic range can be reached at the same loading conditions to 6.61 kN and 5.06, respectively.

**Figure 20.**
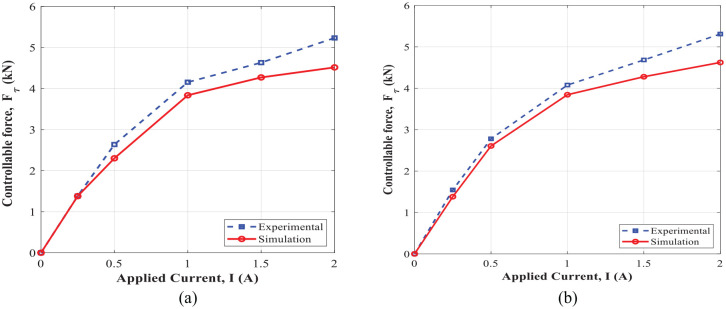
Variation of the controllable damping force with applied currents: (a) excitation frequency of 1 Hz and amplitude of 1 mm and (b) excitation frequency of 2 Hz and amplitude of 1 mm.

**Figure 21.**
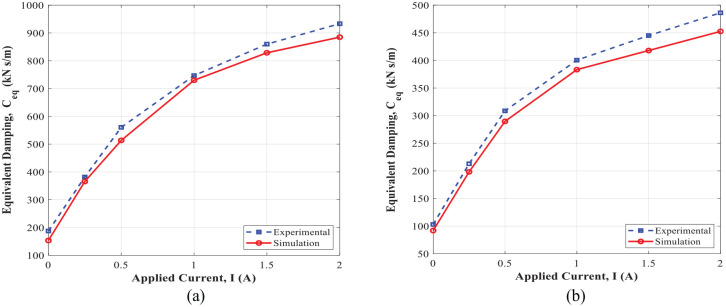
Variation of the damping coefficient with applied currents: (a) excitation frequency of 1 Hz and amplitude of 1 mm and (b) excitation frequency of 2 Hz and amplitude of 1 mm.

**Figure 22.**
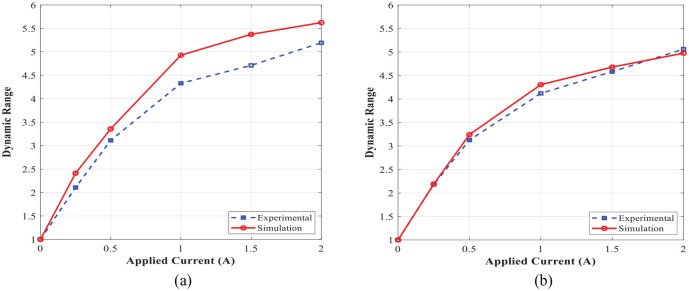
Variation of the dynamic range of the novel MR damper with respect to the applied current: (a) excitation frequency of 1 Hz and amplitude of 1 mm and (b) excitation frequency of 2 Hz and amplitude of 1 mm.

Figure 20 shows the comparison of the controllable damping force obtained experimentally and using an analytical approach (equation (15)), under varied applied currents and different excitation conditions. It is noted that the yield stress in the active region of annular and radial MR fluid gaps ( $\tau_{\mathrm{ya}(r)}\left(B\right))$ was obtained using equation (18). The yield stress depends on the magnetic flux density, as obtained using magnetic circuit analysis in equation (28). Figure 21 also illustrates the equivalent damping coefficient $C_{\mathrm{eq}\;}$ evaluated experimentally using the energy dissipation obtained from the enclosed area in force-displacement hysteresis loops (Figures 18 and 19) and also analytically using equation (16). The variation of the MR damper dynamic range evaluated experimentally and analytically using equation (17) with respect to applied current under different excitation conditions are also shown in Figure 22. Results show very good agreement between the simulation and experimental results and confirm the significant field-dependent controllability of the damper at different operation conditions. The slight deviation between the experimental and simulation modeling was observed. This may be partly attributed to the quasi-static modeling based on the Bingham plastic fluid behavior. Since the Bingham plastic model is not able to consider other factors such as the inertia effect, fluid compressibility, non-uniform distribution of the magnetic flux, and temperature effects.

## 7. Conclusion

In this study, a compact MR fluid damper with a bypass valve containing radial and annular flow gaps has been modeled, optimally designed and experimentally tested, and validated for applications in off-road tracked vehicles. Using the Bingham plastic model, a mathematical model was formulated for determining the total damping force as a function of the MR valves’ geometrical parameters, piston velocity, and yield stress. A magnetic circuit analysis was performed to evaluate the induced magnetic flux density within the annular and radial gaps of the MR valve. A FE simulation using FEMM software was also performed to further realize the distribution of magnetic flux density within the gap. A relatively well agreement between the two magnetic circuit analyses was observed. Subsequently, a formal design optimization problem was formulated using the magnetic circuit analysis in order to maximize the MR damper’s dynamic range. The optimally designed MR damper has been fabricated considering the design requirements and experimentally tested to evaluate the damper performance under different applied currents and excitation conditions. Results showed very good agreements between simulation and experimental results for both magnetic circuit and damper performance (dynamic range and damping force). Under applied current of 1.5 A, the theoretical design optimization and experimental results revealed large damping forces of 7.41 and 5.9 kN, respectively, as well as high dynamic ranges of 6.7 and 4.5, correspondingly, under piston velocity of 12.5 mm/s. The prototyped MR damper can be effectively employed for vibration control applications, particularly, in off-road tracked vehicles.
